# *In Vivo* Generation of Immature Inner Hair Cells in Neonatal Mouse Cochleae by Ectopic Atoh1 Expression

**DOI:** 10.1371/journal.pone.0089377

**Published:** 2014-02-20

**Authors:** Zhiyong Liu, Jie Fang, Jennifer Dearman, Lingli Zhang, Jian Zuo

**Affiliations:** 1 Department of Developmental Neurobiology, St. Jude Children's Research Hospital, Memphis, Tennessee, United States of America; 2 Integrated Program In Biomedical Sciences, University of Tennessee Health Science Center, Memphis, Tennessee, United States of America; Instituto de Medicina Molecular, Portugal

## Abstract

Regeneration of auditory hair cells (HCs) is a promising approach to restore hearing. Recent studies have demonstrated that induced pluripotent stem cells/embryonic stem cells or supporting cells (SCs) adjacent to HCs can be converted to adopt the HC fate. However, little is known about whether new HCs are characteristic of outer or inner HCs. Here, we showed *in vivo* conversion of 2 subtypes of SCs, inner border cells (IBs) and inner phalangeal cells (IPhs), to the inner HC (IHC) fate. This was achieved by ectopically activating Atoh1, a transcription factor necessary for HC fate, in IBs/IPhs at birth. Atoh1+ IBs/IPhs first turned on Pou4f3, another HC transcription factor, before expressing 8 HC markers. The conversion rate gradually increased from ∼2.4% at 1 week of age to ∼17.8% in adult. Interestingly, new HCs exhibited IHC characteristics such as straight line–shaped stereociliary bundles, expression of Fgf8 and otoferlin, and presence of larger outward currents than those of outer HCs. However, new HCs lacked the terminal differentiation IHC marker vGlut3, exhibited reduced density of presynaptic Cbtp2 puncta that had little postsynaptic GluR2 specialization, and displayed immature IHC outward currents. Our results demonstrate that the conversion rate of IBs/IPhs *in vivo* by Atoh1 ectopic expression into the IHC fate was higher and faster and the conversion was more complete than that of the 2 other SC subtypes underneath the outer HCs; however, these new IHCs are arrested before terminal differentiation. Thus, IBs/IPhs are good candidates to regenerate IHCs *in vivo*.

## Introduction

Unlike nonmammalian vertebrates such as birds and fish, mammals cannot regenerate auditory sensory hair cells (HCs) upon damage, which can result in permanent hearing loss [1], [2]. Studies on HC regeneration in chickens suggest that newly regenerated HCs are derived from supporting cells (SCs), which are adjacent to HCs and share common progenitors during development [3], [4]. In birds, SCs can proliferate and transdifferentiate into HCs after HC damage, and during this process the key gene *Atoh1* is upregulated, which encodes a transcription factor required for HC development [5]–[7].

**Figure 1:**
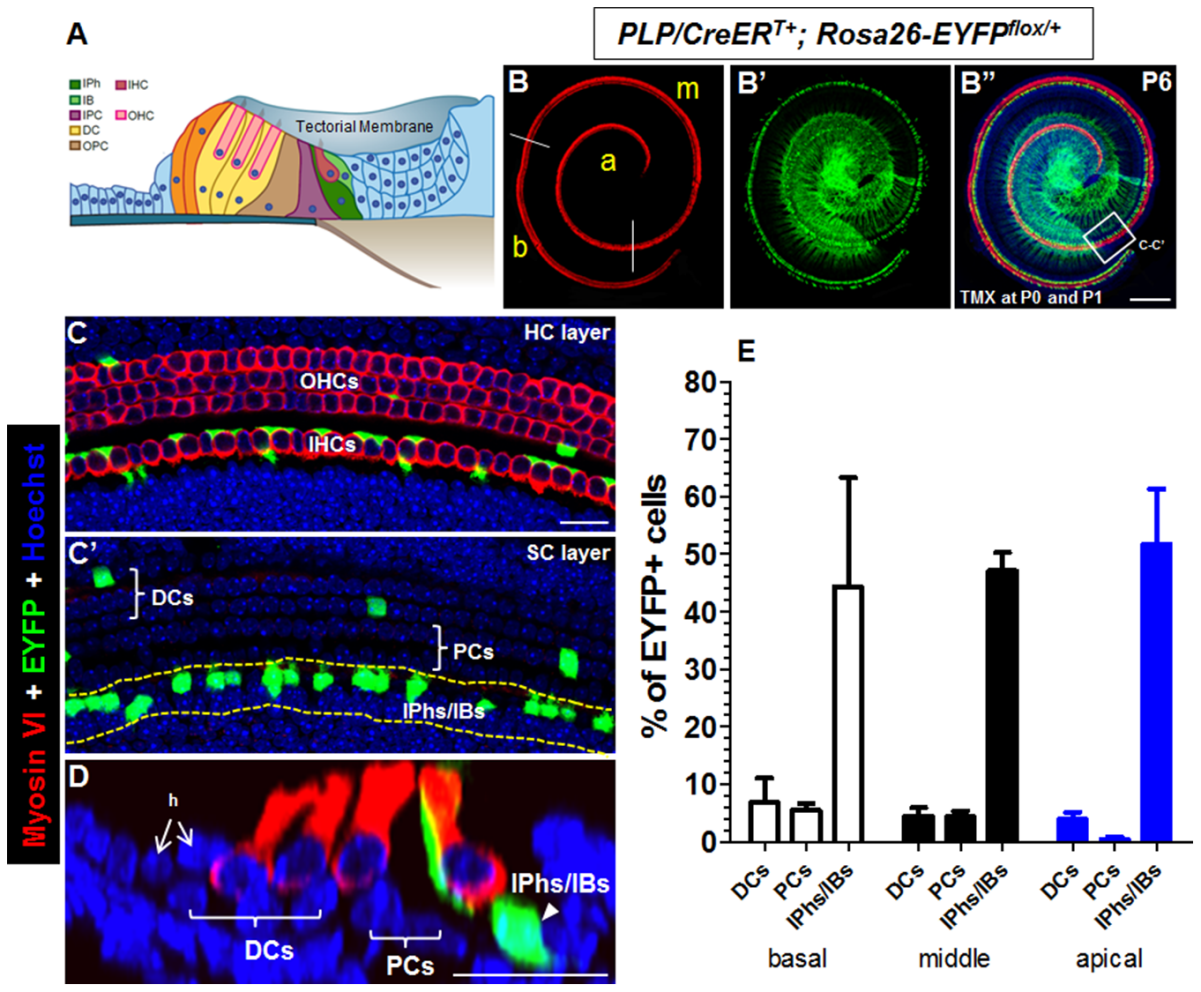
Characterization of Cre activity in *PLP/CreER^T+^; Rosa26-EYFP^flox/+^* mice. (A) Scheme of the neonatal mouse organ of Corti. (B–B’’) Whole-mount double staining of Myosin VI and EYFP. Cochleae were dissected from *PLP/CreER^T+^; Rosa26-EYFP^flox/+^* mice given tamoxifen at P0 and P1 and analyzed at P6. (C–C') High-magnification images of the area within the square in (B’’) at the HC layer (C) and the SC layer (C'). (D) Confocal optical image of the cochlear sample that was double labeled with Myosin VI and EYFP. (E) Percentage of EYFP+ populations among different SC subtypes. b: basal turn; m: middle turn; a: apical turn. h: Hensen's cells; DCs: Deiters' cells; PCs: pillar cells; OPC: Outer Pillar Cell; IPC: Inner Pillar Cell; IBs/IPhs: inner border cells/inner phalangeal cells. Scale bars: 200 µm (B’’); 20 µm (C–D).

The mammalian cochlear sensory epithelium, the organ of Corti, contains 1 row of inner hair cells (IHCs), 3 rows of outer hair cells (OHCs), and a heterogeneous population of surrounding SCs: from medial to lateral, inner border cells (IBs), inner phalangeal cells (IPhs), pillar cells (PCs), Deiters' cells (DCs), and Hensen's cells (Fig. 1A). In *in vivo* studies, ectopic expression of Atoh1 in SCs has led to successful regeneration of HCs in the mammalian cochlea [8]–[10]. However, the exact cell fate of regenerated HCs, that is, whether they become IHCs or OHCs, remains unclear.

It is particularly important to regenerate HCs that can further differentiate into the IHC lineage, because IHCs are normally innervated by 90% of cochlear neurons and are true sensory HCs that are essential for hearing [11]. In the current study, we hypothesized that IBs/IPhs are good candidates for the regeneration of IHCs. They are directly underneath the IHCs and distributed medial to PCs and DCs in the SC layer, thus having a geographic advantage to potentially replace the damaged IHCs. We found that after targeted ectopic Atoh1 induction in IBs/IPhs at postnatal day 0 (P0) and P1, they were converted into the IHC fate *in vivo*, further along the HC differentiation lineage than that described in previous studies. The conversion was faster and more complete than that of PCs and DCs underneath OHCs reported previously [8]. Moreover, the conversion involved activation of Pou4f3, a target of Atoh1, but unlikely involved de-differentiation into the progenitor/stem cell state, before turning on other HC markers. However, new IHCs remained molecularly and electrophysiologically immature.

## Results

### Targeted Ectopic Atoh1 Expression in Neonatal IPhs/IBs

Previous characterization of *PLP/CreER^T+^; Rosa26-LacZ^flox/+^* mice has shown that Cre activity is limited to IBs/IPhs inside the organ of Corti when induced at various postnatal ages [12]. We also independently analyzed *PLP/CreER^T+^; Rosa26-EYFP^flox/+^* experimental mice (*n* = 3, mice number) and control *Rosa26-EYFP^flox/+^* (*n* = 3) injected with tamoxifen at P0 and P1 and analyzed at P6 (Fig. 1B–D). Either left or right cochleae were analyzed of each mouse. Inside the organ of Corti, most of the EYFP-traced cells were IBs/IPhs, but a small fraction was PCs and DCs. No EYFP+ HCs were observed in both experimental and control group by scanning the entire cochlear HCs by confocal microscope. Because there is no difference among different cochlear turns, we grouped 3 turns together and normalized EYFP+ cells to the total number of cells for each SC subtype. Approximately 47.7% of IBs/IPhs, 3.4% of PCs, and 5.2% of DCs were EYFP+ (Fig. 1E). *CAG-loxP-stop-loxP-Atoh1-HA+* mice (hereafter designated as *Atoh1-HA+*) were described in our previous study [8]. FISH analysis (Fig. S1) and genomic Southern blot analysis using a *Xba*I restriction digest showed that this transgenic Atoh1-HA+ line has more than 50 copies of the Atoh1-HA transgene in a single integration event located on chromosome 10 (band 10A3–4).

Next, we crossed heterozygous *PLP/CreER^T+^* with Atoh1-HA+ mice, and in each littermate *PLP/CreER^T+^; Atoh1-HA+* mice were used as the experimental group and *Atoh1-HA+* littermates (without *PLP/CreER^T^*) as the control group. Both groups received identical treatments with tamoxifen at P0 and P1 and were analyzed at various ages as described below. Ectopic Atoh1 expression in the small fraction (3%–5%) of PCs and DCs were identical to those observed in our previous study [8].

### Ectopic Atoh1 Expression Rapidly Converts Neonatal IBs/IPhs into HCs

**Figure 2:**
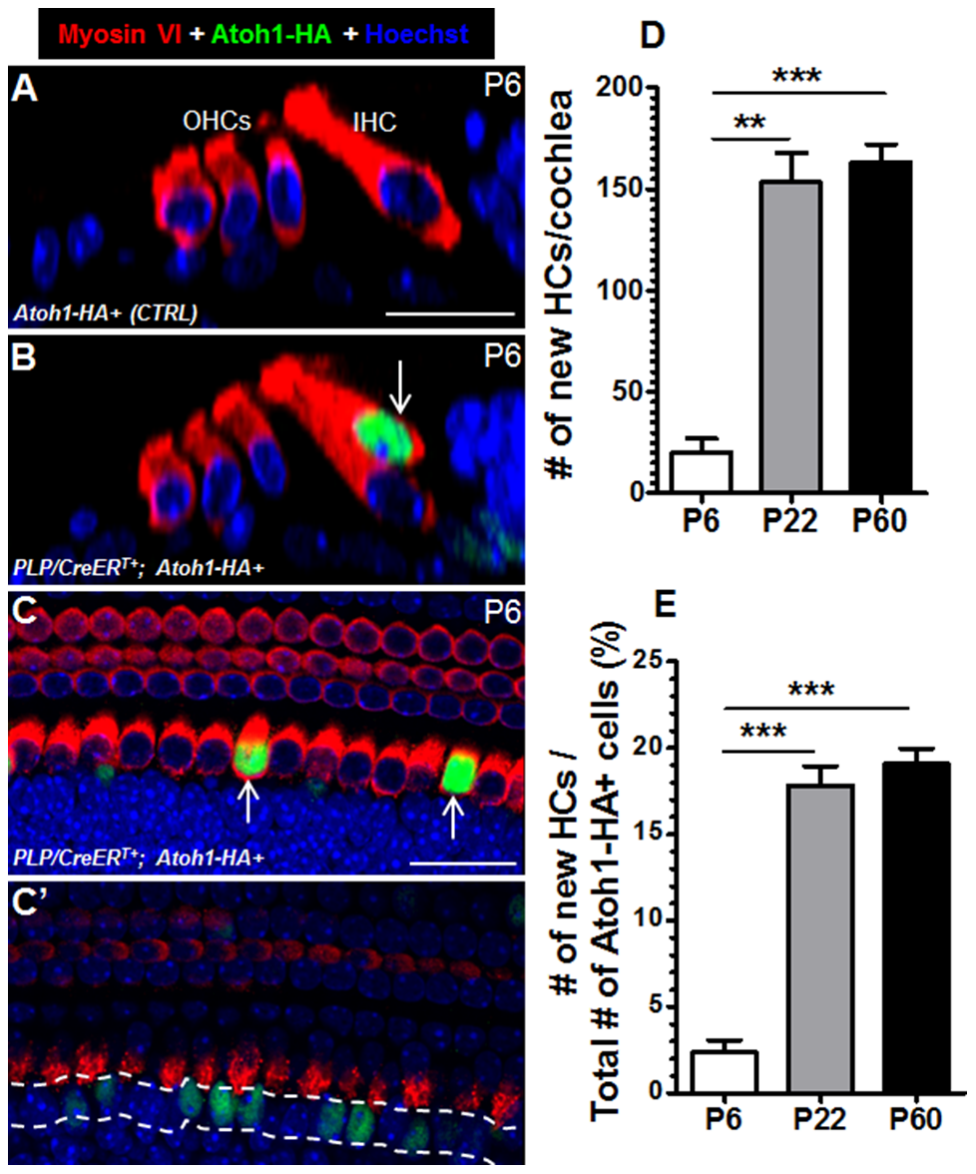
Conversion of neonatal IBs/IPhs into HCs by ectopic Atoh1-HA expression. (A–B) Optical images from the colabeling of Myosin VI and Atoh1-HA in the cochleae of control (A) and *PLP/CreER^T+^; Atoh1-HA+* mice (B) at P6. The arrow in (B) points to a new HC. (C–C') A whole-mount confocal image taken at the HC layer (C) and the IBs/IPhs layer (C') from a *PLP/CreER^T+^; Atoh1-HA+* cochlea at P6. Many Atoh1-HA+ IBs/IPhs (green cells within the dashed line) did not turn on Myosin VI. (D) Quantification of the total new HCs based on Atoh1-HA and Myosin VI co-expression in the entire cochleae at P6, P22, and P60 (**P<0.01, ***P<0.001, *n* = 3). (E) The reprogramming efficiency was calculated by normalizing the new HCs to the total Atoh1-HA+ IBs/IPhs/new HCs at P6, P22, and P60 (***P<0.001, *n* = 3). Scale bar: 20 µm.

**Figure 3:**
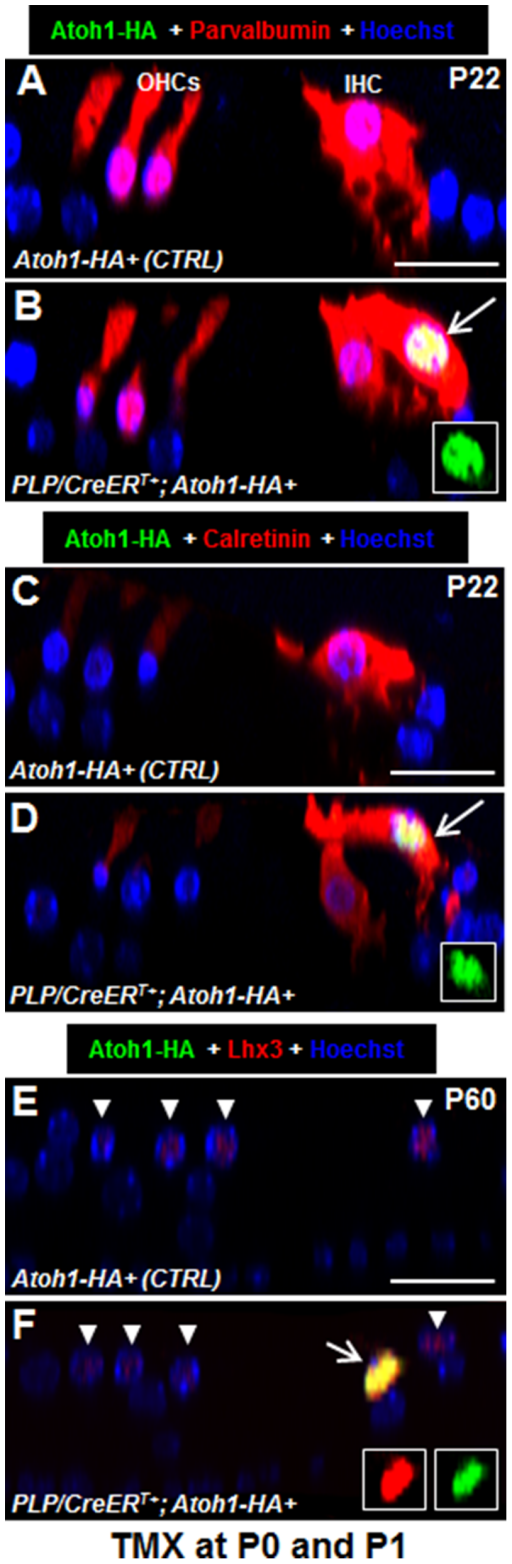
New HCs express HC markers. (A–B) Optical confocal image of Atoh1-HA and parvalbumin costaining in control (A) and *PLP/CreER^T+^; Atoh1-HA+* (B) mice at P22. (C–D) Optical confocal image of Atoh1-HA and calretinin costaining in control (C) and *PLP/CreER^T+^; Atoh1-HA+* (D) mice at P22. (E–F) Optical confocal image of Atoh1-HA and Lhx3 costaining in control (E) and *PLP/CreER^T+^; Atoh1-HA+* (F) mice at P60. Arrows in (B), (D), and (F) point to new HCs. The inset in (B), (D), and (F) shows a single-channel signal for Atoh1-HA and/or Lhx3. Arrow heads in (E) and (F) represent endogenous OHCs and IHCs. OHCs: outer hair cells; IHC: inner hair cell. Scale bar: 20 µm.

**Figure 4:**
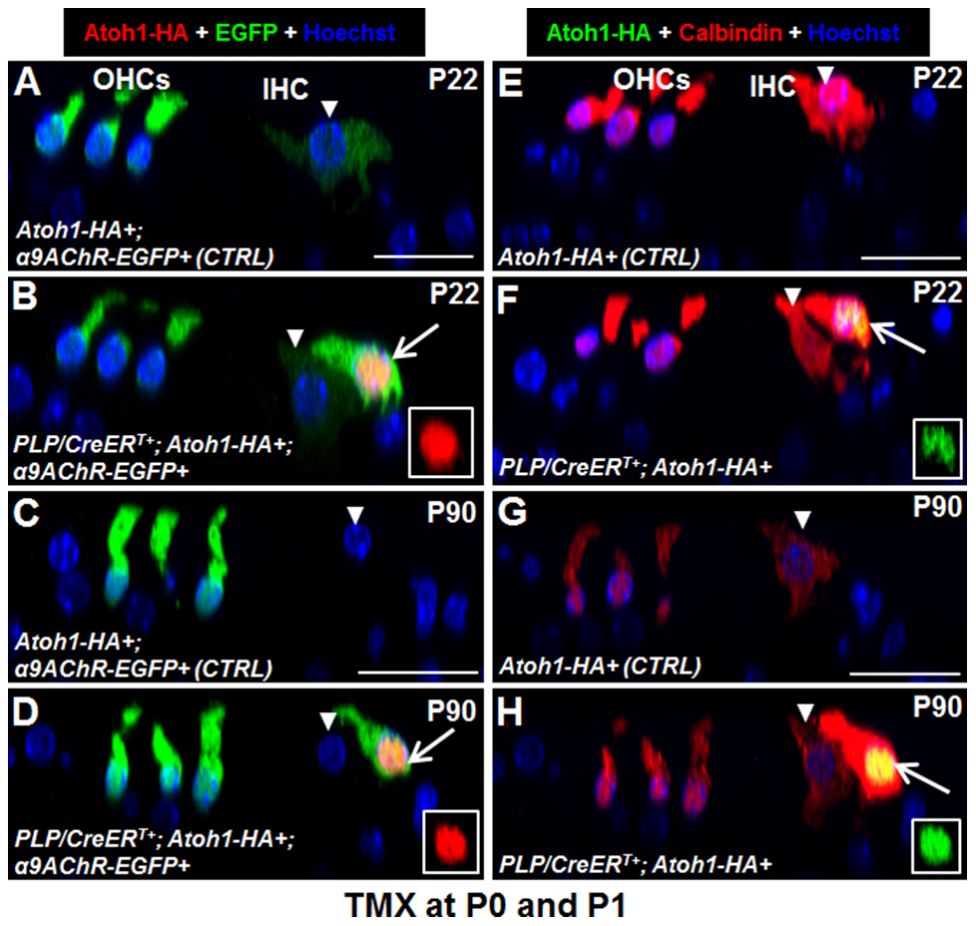
New IHCs cannot downregulate α9AChR and calbindin at older ages. (A–D) Double staining of Atoh1-HA and EGFP in control (A, C) and *PLP/CreER^T+^; Atoh1-HA+; α9AChR-EGFP+* (B, D) mice at P22 (A, B) and P90 (C, D). (E–H) Colabeling of Atoh1-HA and calbindin in control (E, G) and *PLP/CreER^T+^; Atoh1-HA+* (F, H) mice at P22 (E, F) and P90 (G, H). Arrows in (B), (D), (F), and (H) represent new IHCs. The inset in (B), (D), (F), and (H) shows a single-channel signal for Atoh1-HA. Arrow heads in (A–H) represent endogenous IHCs. OHCs: outer hair cells; IHC: inner hair cell. Scale bar: 20 µm.

HA antibody was used to visualize and trace ectopic Atoh1 expression, and samples were analyzed at P4, P6, P22, and P60. In the control group, there were no Atoh1-HA+ cells or ectopic HCs at all ages (Fig. 2A), confirming that our Atoh1-HA+ transgene was not expressed without Cre-mediated recombination. However, starting at P6, *PLP/CreER^T+^; Atoh1-HA+* experimental mice had Atoh1-HA+ cells that coexpressed myosin VI (Fig. 2B–C), an early HC-specific marker [13]. Intriguingly, all Atoh1-HA+/Myosin VI–negative IBs/IPhs remained in the SC layer (Fig. 2C'). At P6, 830±40 (*n* = 3) Atoh1-HA+ cells were observed across the entire length of the cochlea. Of the Atoh1-HA+ cells, 20±6 (*n* = 3) cells became Myosin VI+ (Fig. 2D), making up 2.4%±0.7% of the total Atoh1-HA+ cells (Fig. 2E). There was no difference in the number of Atoh1-HA+/Myosin VI+ cells among different cochlear turns. The Atoh1-HA+/Myosin VI+ cells were adjacent to the endogenous IHCs (both medial and lateral). However, the number of Atoh1-HA+/Myosin VI+ cells present across the entire cochlea at P22 was 153±14 (*n* = 3), which was much higher than that at P6 (20±6; Fig. 2D). The Atoh1-HA+/Myosin VI+ cells comprised 17.8%±1.1% of the total Atoh1-HA+ cells at P22 (Fig. 2E). The number of Atoh1-HA+/Myosin VI+ cells did not further increase at P60 (Fig. 2D). At P22, P60 and P90, besides Myosin VI and Myosin VIIa, Atoh1-HA+ cells expressed 5 additional HC markers: parvalbumin, calretinin, α9AChR, calbindin, and Lhx3 (Figs. 3 and 4), supporting the conclusion that Atoh1 converted IBs/IPhs into HCs. We defined Atoh1-HA+ cells that expressed these HC markers as new HCs.

**Figure 5:**
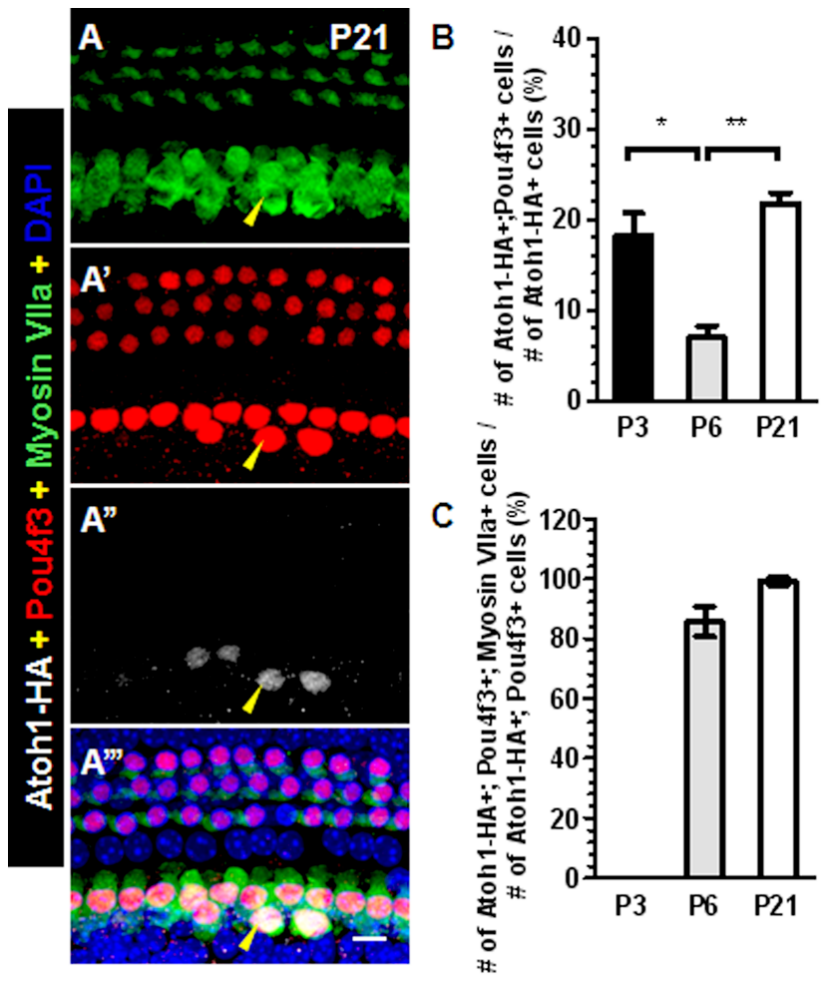
Pou4f3 is expressed, prior to other HC markers, in the new HCs. (A–A’’’) Triple staining image of Myosin VIIa, Pou4f3 and Atoh1-HA in cochlea of *PLP/CreER^T+^; Atoh1-HA+* mice. Arrowheads in (A-A’’’) point to a new HC expressing Atoh1-HA, Pou4f3 and Myosin VIIa. (B) Percentages of Atoh1-HA+/Pou4f3+ cells among total Atoh1-HA+ cells at various ages. * p<0.05 (*n* = 3), ** p<0.01 (*n* = 3). (C) Percentages of Atoh1-HA+/Pou4f3+/Myosin VIIa+ new HCs among total Atoh1-HA+/Pou4f3+ cells at various ages. Note that Pou4f3 is expressed in ∼20% Atoh1-HA+ cells at P3 prior to Myosin VII expression and that at P6 and P21, there were Atoh1-HA+/Pou4f3+ cells that had yet to express Myosin VII. Scale bar: 10 µm.

To gain further molecular insights into the cell fate conversion process, we determined whether cell fate conversion involves, to some degree, dedifferentiation of IBs/IPhs to the progenitor or stem cell state or exclusively direct transdifferentiation from IBs/IPhs to HCs. We performed co-labeling of Atoh1-HA and several early progenitor/stem cell markers such as NeuroD1 [14], Neurogenin 1 or Ngn1 [14] and Oct4 [15], which are expressed in the otic placode cells, and the embryonic stem cell (ES) marker Nanog [16], [17]. Co-staining was performed at P3 (*n* = 3) and P6 (*n* = 3) during the initial phase of cell fate conversion. None of the Atoh1-HA+ cells expressed these markers at P3 (Fig. S2) or P6 (data not shown), supporting the notion that de-differentiation to the progenitor/stem cell state is unlikely involved in the conversion. Next, we determined whether IBs/IPhs followed the normal process of HC development. We performed triple immunostaining of Atoh1-HA, Pou4f3, and Myosin-VIIa. Pou4f3 (also known as Brn3c) is an HC transcription factor whose expression starts in a basal-to-apical gradient around embryonic day (E) 14.5 [18] but is maintained at all postnatal ages [19]. All new HCs (Atoh1-HA+/Myosin VIIa+) expressed Pou4f3 at P21 (Fig. 5A-A’’’). In addition, among the total Atoh1-HA+ cells (*n* = 3 at each age), we found that ∼18.1% at P3, ∼6.9% at P6, and ∼21.7% at P21 expressed Pou4f3 (Fig. 5B). The decreased percentile at P6 might be due to the faster increment of total Atoh1-HA+ cells (between P3 and P6) than the activation of Pou4f3. Activation of Pou4f3 in Atoh1-HA+ IBs/IPhs at P3 when no new HCs were observed suggests that Pou4f3 is needed for cell fate conversion. Interestingly, among the Atoh1-HA+/Pou4f3+ cells, ∼85% of them at P6, and ∼99% of them at P21 were Atoh1-HA+/Pou4f3+/Myosin VIIa+ and became new HCs (Fig. 5C), which is much higher than ∼2.4% and ∼17.8% (percentile of new HCs among total Atoh1-HA+ cells at P6 and P22, respectively). This supports that Pou4f3 and Atoh1 together are more competent to convert IBs/IPhs into HCs than Atoh1 alone. Finally, to confirm that Pou4f3 is induced by Atoh1 and Pou4f3 expression precedes cell fate conversion, we also examined Atoh1-HA+ cells in the OHC area where a few PCs/DCs were also targeted in *PLP/CreER^T+^; Atoh1-HA+* mice. Consistent with the results from our previous study showing that it takes 22–60 days for DCs/PCs to become HCs after ectopic Atoh1 induction at P0 and P1 [8], the Atoh1-HA+ PCs/DCs had turned on Pou4f3, but not yet Myosin-VIIa at P21, suggesting that cell fate conversion occurs later than Pou4f3 expression (data not shown). Together, these results support the conclusion that ectopic Atoh1 activates Pou4f3, and they might promote cell fate conversion and the expression of multiple HC-specific markers, either independently or synergistically.

**Figure 6:**
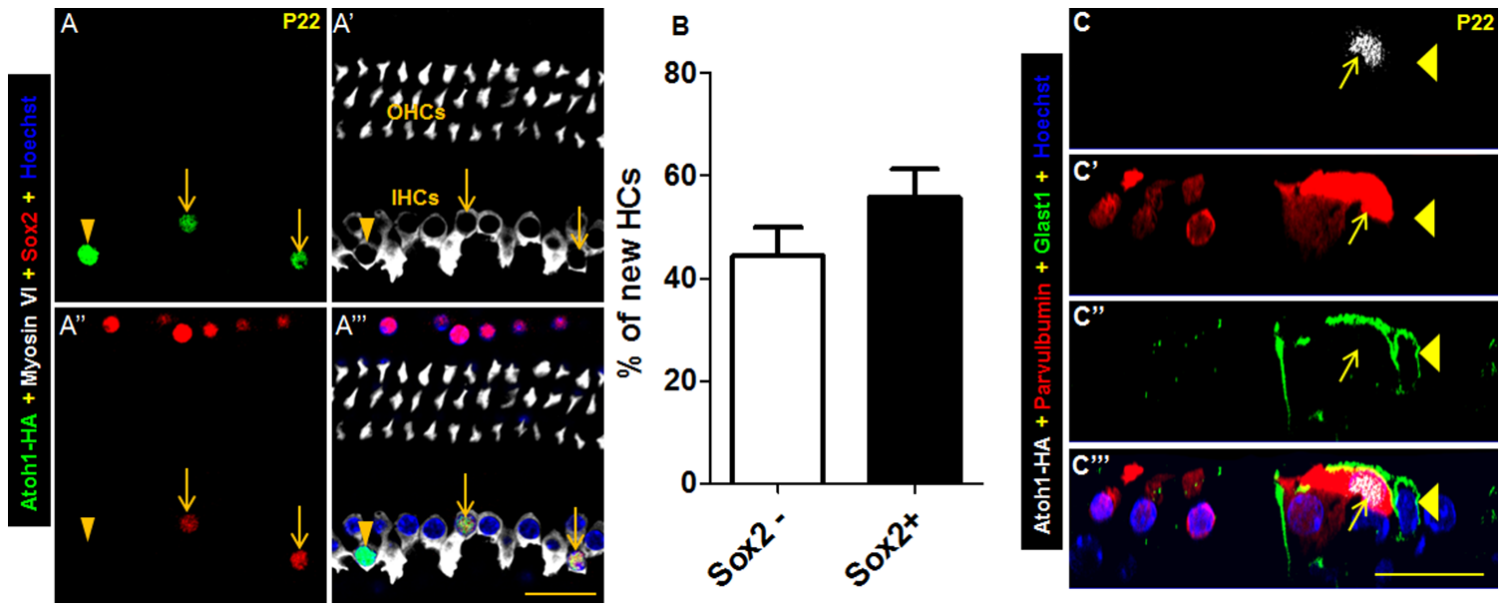
Heterogeneous Sox2 expression and absence of Glast1 in new HCs. (A–A’’’) Whole-mount triple staining of Atoh1-HA, myosin VI, and Sox2 in cochleae of *PLP/CreER^T+^; Atoh1-HA+* mice at P22. Arrows point to new HCs that maintained Sox2 expression. The arrowhead represents the new HC without Sox2 expression. (B) Percentages of Sox2-positive and Sox2-negative new HCs. OHCs: outer hair cells; IHC: inner hair cell. (C–C’’’) Triple staining of Atoh1-HA, parvalbumin, and Glast1 in cochleae of *PLP/CreER^T+^; Atoh1-HA+* mice at P22. Arrows show the new HC that did not express Glast1. Arrowheads point to a Glast1+ IB/IPh cell that wraps the new HC. There was no overlap of parvalbumin and Glast1 signals at the top of the new HC membrane. Scale bar: 20 µm.

At P22, approximately 57% of new HCs retained the expression of Sox2 (Fig. 6A–B, (*n* = 3), a cochlear SC-specific marker at postnatal ages [20], indicating that there are differences in the extent of conversion among new HCs and supporting the potential regulation of Sox2 expression by persistent Atoh1 expression [21], [22]. To determine whether other IB/IPh markers were still maintained at later ages of new HCs, we analyzed in new HCs at P21 the expression of Glast1, a glutamate-aspartate transporter that is normally weakly expressed in neonatal IBs/IPhs but highly expressed in adult IBs/IPhs and is responsible for glutamate uptake at IHC afferent synapses [23], [24]. New HCs at P21 that were Atoh1-HA+; parvalbumin+ were surrounded by Glast1+ processes of IBs/IPhs but were themselves negative for Glast1 (Fig. 6C-C’’’). Therefore, while Sox2 expression might suggest a hybrid state of the new nascent HCs, they adopted many HC characteristics in their initial transdifferentiation state and eventually lost Glast1 expression in their final differentiation state.

### New HCs Adopt the IHC Fate

**Figure 7:**
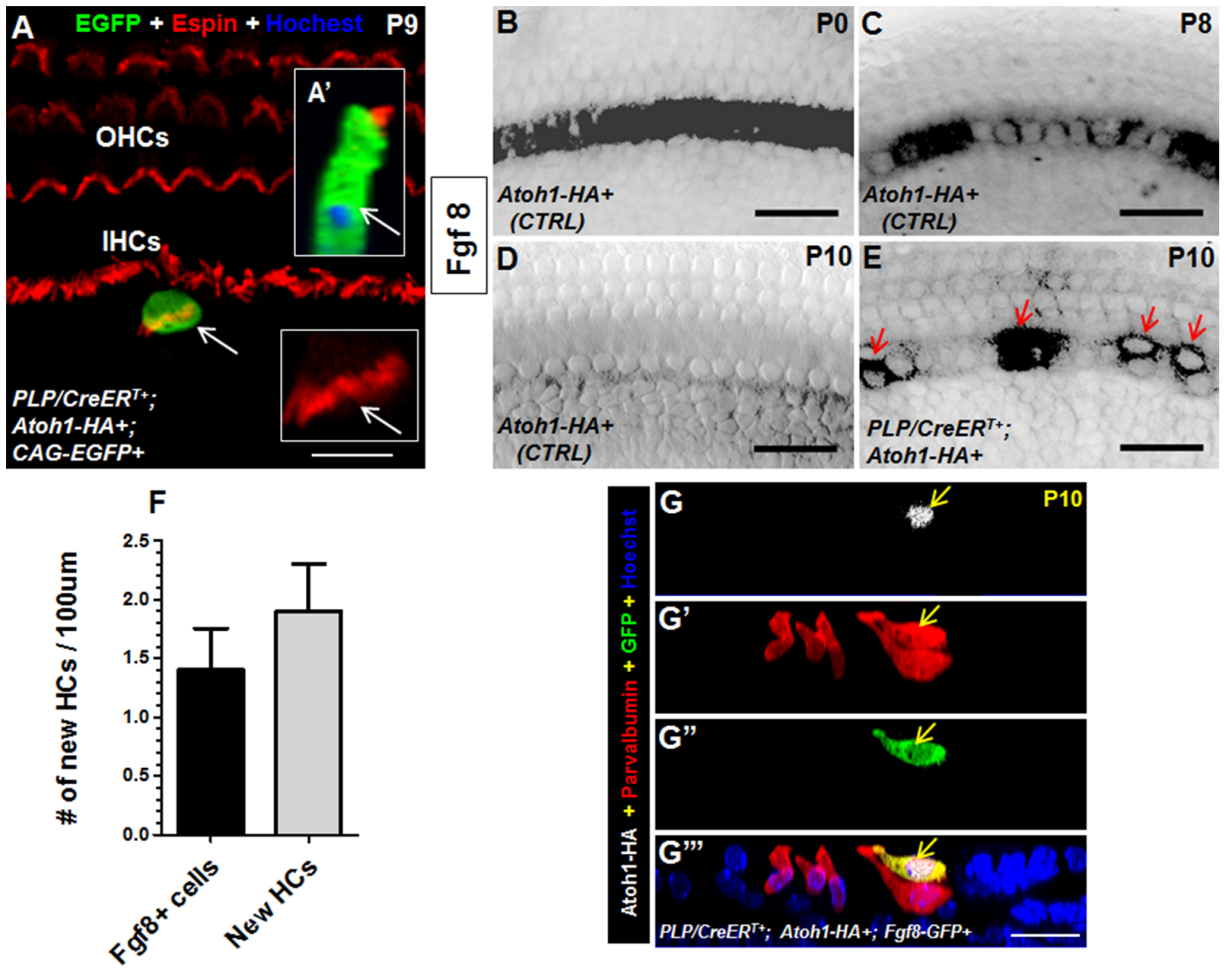
New HCs adopt the IHC fate. (A–A') Confocal image of EGFP and Espin double staining at the top of the HC layer. Cochleae were dissected from *PLP/CreER^T+^; Atoh1-HA+; CAG-EGFP+* mice given tamoxifen at P0 and P1 and analyzed at P9. Arrows show the same new HC with the straight line–shaped stereociliary bundles. (A') The same new HC viewed at the confocal YZ angle. (B–D) Whole-mount Fgf8 *in situ* hybridization of control cochleae at P0 (B), P8 (C), and P10 (D). (E) Whole-mount Fgf8 *in situ* hybridization in the cochlea from a *PLP/CreER^T+^; Atoh1-HA+* mouse that was given tamoxifen at P0 and P1 and analyzed at P10. Arrows point to Fgf8+ cells. (F) Comparison of cell numbers from *in situ* hybridization of Fgf8+ cells and new HCs (Myosin VI+/Atoh1-HA+) at P10 showed no differences. (G–G’’’) Triple staining of Atoh1-HA, parvalbumin, and GFP in the cochlea of *PLP/CreER^T+^; Atoh1-HA+; Fgf8-GFP+* mice at P10. Arrows target the new HC expressing Fgf8. OHCs: outer hair cells; IHCs: inner hair cells. Scale bars: 10 µm (A), 20 µm (B–E, G’’’).

We next determined whether the new HCs were phenotypically IHCs or OHCs by using morphological characteristics and IHC-specific markers. The ciliary structure on the apical surface, referred to as the stereociliary bundle, is a characteristic of HCs. OHCs have V- or W-shaped stereociliary bundles whereas IHCs have straight-line–shaped stereociliary bundles, which allowed us to differentiate whether new HCs were IHCs or OHCs. To trace both the nuclei and cytoplasm of Cre+ IBs/IPhs with EGFP, we crossed *CAG-loxP-stop-loxP-EGFP+* (hereafter *CAG-EGFP+*) mice [25] with *PLP/CreER^T+^; Atoh1-HA+* mice. The *PLP/CreER^T+^; Atoh1-HA+; CAG-EGFP+* mice were treated with tamoxifen at P0 and P1 and analyzed at P9. The actin-binding protein Espin was used to visualize stereociliary bundles [26]. The expression of Espin at the top surface of EGFP+ cells (derived from Cre+ IBs/IPhs) confirmed the formation of stereociliary bundles at P9 in the new HCs (Fig. 7A-A'). Also, these EGFP+/Espin+ new HCs were Atoh1-HA+ in the nucleus. The stereociliary bundle morphology of all such new HCs (15±5, *n* = 3) was very similar to that of endogenous IHCs but not outer HCs at P9.

Next, we determined whether the new HCs expressed Fibroblast growth factor 8 (Fgf8), which is specifically expressed in IHCs and plays key roles in cochlear development [18], [27], [28]. *In situ* hybridization analysis with the Fgf8 probe confirmed the unique expression of Fgf8 in control IHCs at P0 (Fig. 7B), which is consistent with previous reports [18], [27], [28]. Also, Fgf8 expression decreased at P8 and became undetectable at P10 in control cochleae (Fig. 7C, D). In contrast, in experimental *PLP/CreER^T+^; Atoh1-HA+* cochleae, Fgf8-expressing cells at P10 were located medially or laterally adjacent to endogenous IHCs (arrows in Fig. 7E). Furthermore, the numbers of Fgf8-expressing cells and new HCs at P10 were similar, suggesting that new HCs were IHCs (Fig. 7F). To further confirm that new HCs express Fgf8 at a single-cell resolution, we bred conditional *Fgf8-GFP* mice with *PLP/CreER^T+^; Atoh1-HA+*mice. The *Fgf8-GFP* was generated in such a way that GFP was controlled by the endogenous *Fgf8* promoter and 1 copy of the *Fgf8* allele was inactivated (exon 5 is lost) after Cre-mediated recombination [29]. The new HCs that were Atoh1-HA+; parvalbumin+; GFP+ were observed in *PLP/CreER^T+^; Atoh1-HA+; Fgf8-GFP+* mice at P10 (Fig. 7G-G’’’). Of note, the loss of 1 copy of Fgf8 at neonatal ages did not affect the gross development of the cochlea (data not shown). Taken together, our results support the conclusion that new HCs express Fgf8 and are IHCs.

New HCs in the IHC region also expressed calretinin, and its level in the new IHCs was comparable to that in endogenous IHCs (Fig. 3D). We have previously shown that in wild-type mice calretinin is highly expressed in IHCs but very weakly expressed in OHCs at these ages (Fig. 3C) [8]. New HCs in the IHC region did not express the OHC-specific marker prestin [30], [31]. Taken together, our results support the conclusion that ectopic Atoh1 expression destines neonatal IBs/IPhs to the IHC fate and they do not maintain Glast1 expression. Thus, we hereafter define new HCs in the IHC region as new IHCs.

### New IHCs Uptake the FM4-64 Dye and Display Outward Currents

We next characterized the functionality of new IHCs. The initial step of sensing sound by HCs starts at the opening of the mechanosensory transduction (MET) channel located in the stereocilia bundles [32]. Whole-mount cochlear sensory epithelia of *PLP/CreER^T+^; Atoh1-HA+* were exposed transiently (30 second incubation) to FM4-64FX dye at P13, P30, and P90. Uptake of the FM4-64FX dye occurred in Atoh1-HA+ new IHCs at all ages (Fig. S3) [33], [34]. Furthermore, when cochlear samples were treated with the FM4-64FX dye after pre-treatment with 5 mM EGTA or 5 mM BAPTA for 15 min to break the tip links which are critical for MET channel function [35], [36], the intensity of FM4-64FX within Atoh1-HA+ new IHCs was substantially decreased or absent (data not shown). Taken together, our results support the conclusion that the MET channel is present and presumably functional in new IHCs formed in our model.

**Figure 8:**
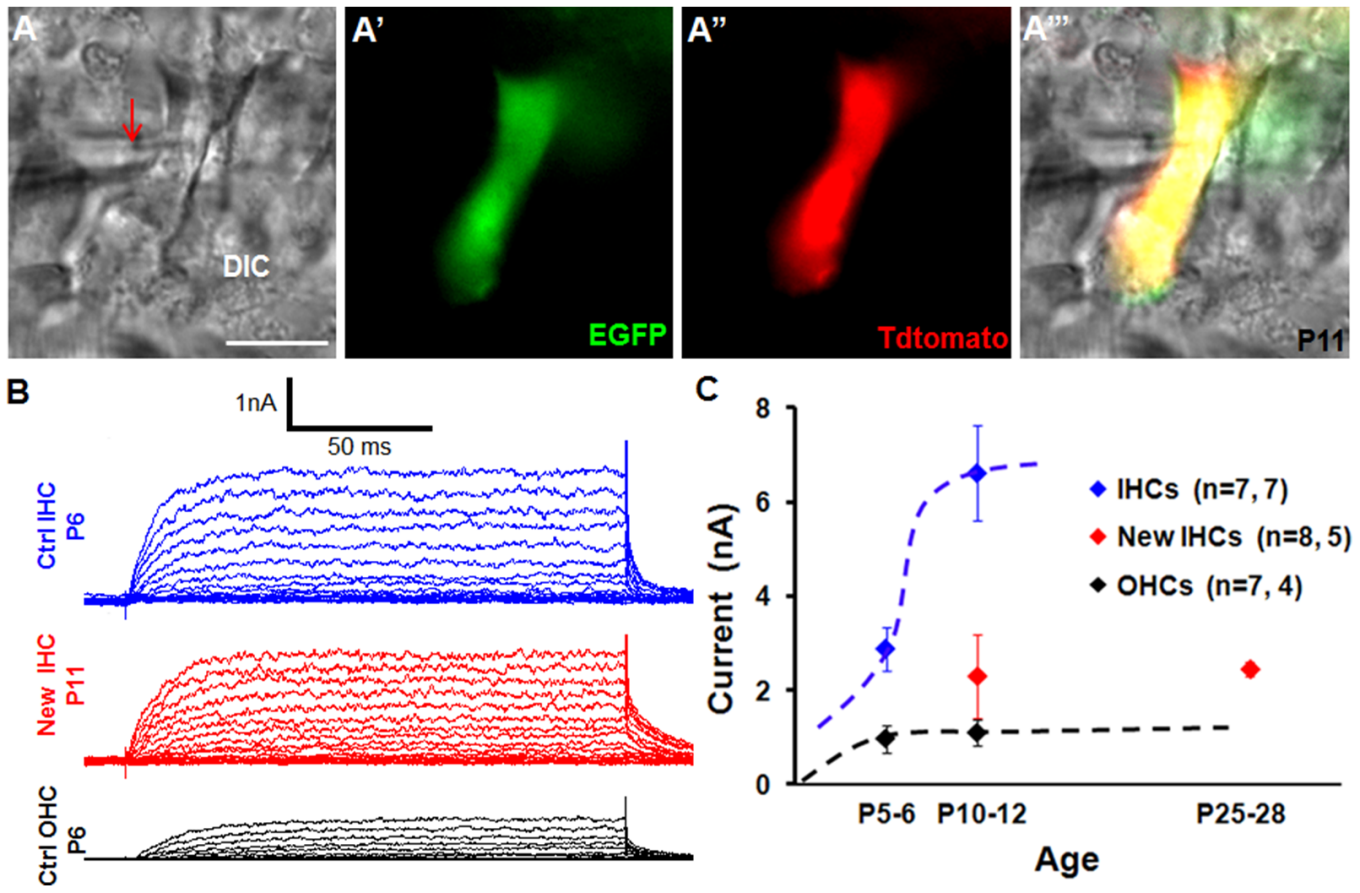
New IHCs display outward current. (A–A’’’) An EGFP+/Tdtomato+ new IHC was patched by the recording electrode (red arrow in A). The cochlea was dissected from the *PLP/CreER^T+^; Atoh1-HA+; Rosa26-CAG-Tdtomato^flox/+^; α9AChR-EGFP+* line. (B) Representative traces of voltage-dependent whole-cell currents from endogenous IHCs at P6 (blue), new IHCs at P11 (red), and endogenous OHCs at P6. Cells were voltage clamped at −84 mV and stepped from −120 to +50 mV in 10-mV increments. (C) Comparison of outward current amplitude of control OHCs (black) and IHCs (blue) and new IHCs (red). *n:* number of cells measured at different ages; Ctrl: control. Scale bars: 10 µm.

Because outward current amplitudes are significantly different between IHCs and OHCs [37], [38], we therefore determined the outward current of new IHCs by whole-cell patch clamp. To distinguish new IHCs from neighboring endogenous IHCs, we analyzed compound *PLP/CreER^T+^; Atoh1-HA+; Rosa26-CAG-tdtomato^flox/+^; α9AChR-EGFP+* mice in which EGFP expression is driven by the promoter of another HC marker α9AChR [39]. New IHCs were tdTomato+ (derived from Cre+ IBs/IPhs) and EGFP+, whereas endogenous IHCs only weakly expressed EGFP (Fig. 8A-A’’’). New IHCs exhibited outward currents under whole-cell voltage clamp at various levels from a holding potential of −84 mV (Fig. 8B). These traces were similar to those of control IHCs under similar conditions. The maximum outward current amplitude of new IHCs reached 2.3±0.9 nA (*n* = 8) at P10–P12 and remained at 2.4±0.2 nA (*n* = 5) at P25–P28 (Fig. 8C). In contrast, in control IHCs, maximum outward current amplitudes increased from 2.9±0.5 nA (*n* = 7) at P5–P6 to 6.6±1.0 nA (*n* = 7) at P10–P12, whereas in control OHCs amplitudes were 1.0±0.1 nA (*n* = 7) at P5–P6 and 1.1±0.3 nA (*n* = 4) at P10–P12 (Fig. 8C). Taken together, our results support the conclusion that new IHCs at P10–P12 behaved similarly to control IHCs at P5–P6 (*P*>0.05) and were significantly different from control OHCs at P4–P6 (*P*<0.05) and P10–P12 (*P*<0.001). However, they could not further improve their outward current amplitude at P25–P28 as control IHCs did at P10–P12 (*P*<0.001).

### New IHCs Are Not Fully Differentiated

**Figure 9:**
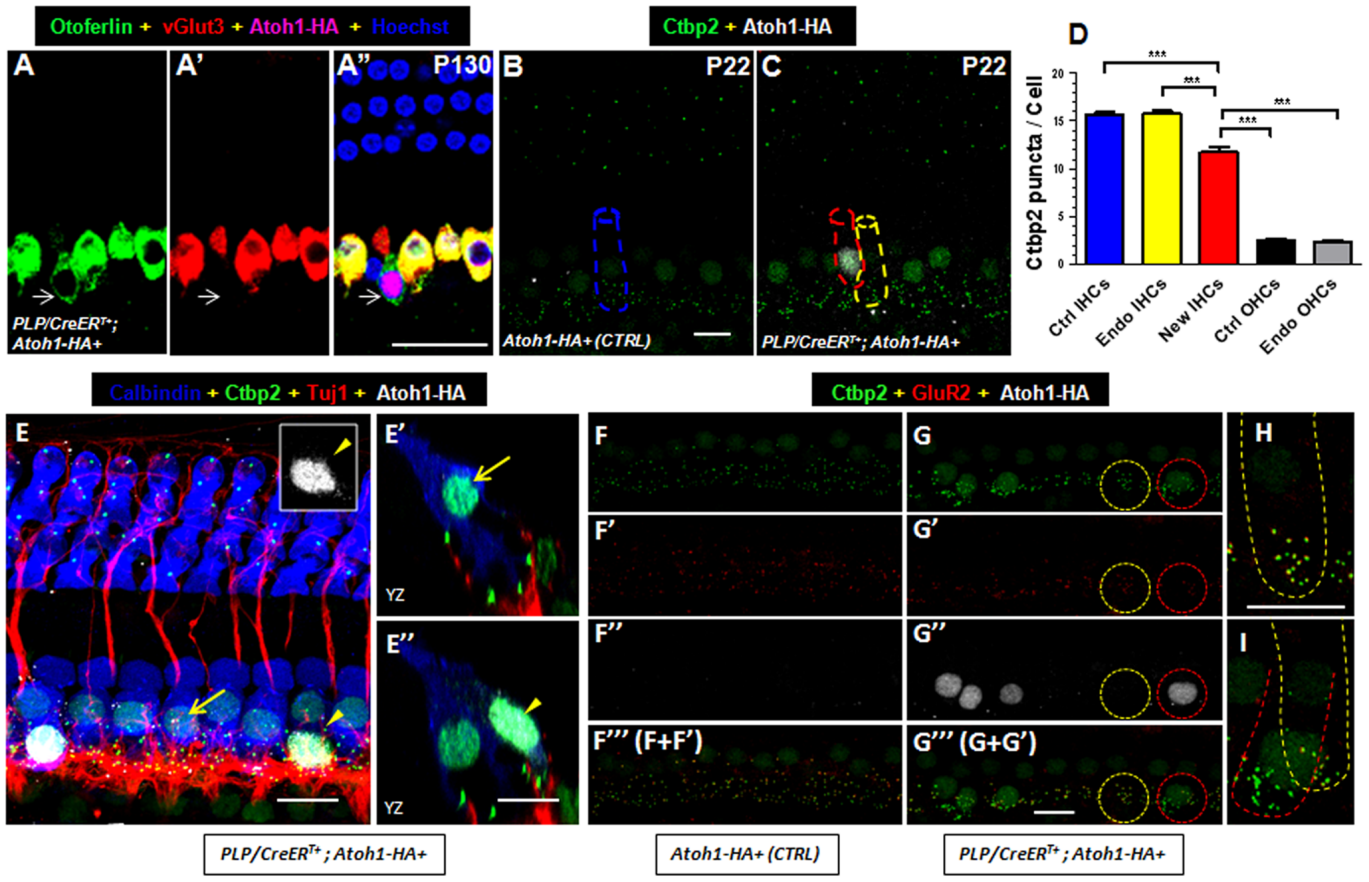
New IHCs are not fully differentiated. (A–A’’) Whole-mount triple staining of otoferlin, vGlut3, and Atoh1-HA in the cochlea of a *PLP/CreER^T+^; Atoh1-HA+* mouse given tamoxifen at P0 and P1 and analyzed at P130. Arrows show the same new IHC that was Atoh1-HA+/otoferlin+ but vGlut3-negative. (B–C) Double staining of Ctbp2 and Atoh1-HA in cochleae of control (B) and *PLP/CreER^T+^; Atoh1-HA+* mice (C). The cell on the red dotted line (C) represents a new IHC, and the cell on the yellow dotted line (C) is an endogenous IHC. On the basis of Myosin VI staining, we determined the contour of the cell (dotted lines in B and C) and measured the density of Ctbp2. (D) Comparison of the number of Ctbp2 puncta per cell among different types of HCs (***P<0.001). (E-E’’) Co-staining of calbindin, Ctbp2, Tuj1, and Atoh1-HA in the cochlea of a *PLP/CreER^T+^; Atoh1-HA+* mouse at P22. Arrows in (E, E') show the same endogenous IHC. Arrowheads in (E, E’’) show the same new IHC. The inset in (E) shows the Atoh1-HA channel of the new IHC only. (E') and (E’’) are images visualized in the confocal YZ plane. (F–I) Triple labeling of Ctbp2, GluR2, and Atoh1-HA in both the control (F-F’’’) and experimental (G-G’’’) groups. The same cell in the red circle is a new IHC in which GluR2 is absent (green spots), whereas the same cell in the yellow circle is the endogenous IHC in which the Ctbp2 and GluR2 are well aligned (yellow spots). The same circled cells in (G-G’’’) were visualized in high magnification images in (H) and (I). Endo: Endogenous; Ctrl: control; IHCs: inner hair cells; OHCs: outer hair cells. Scale bars: 20 µm (A’’), 10 µm (B, E, G’’’).

The failure of new IHCs to further improve their outward current amplitude suggested that they were not fully differentiated. To confirm this possibility, we co-labeled Atoh1-HA, otoferlin, and vGlut3. Otoferlin is a synaptic vesicle protein that is expressed in HCs in the cochlea [40], [41]. vGlut3 is an IHC terminal differentiation marker required for IHC function [40], [42], [43]. Atoh1-HA+/otoferlin+/vGlut3–negative new IHCs were observed in the cochleae of *PLP/CreER^T+^; Atoh1-HA+* mice at P130, whereas endogenous IHCs in *PLP/CreER^T+^; Atoh1-HA+* mice displayed normal vGlut3 expression (Fig. 9A-A’’). Otoferlin is enriched at much higher level in endogenous IHCs than in OHCs at these ages [44], further supporting the conclusion that new HCs adopted the IHC fate. We also confirmed that vGlut3 expression was absent in new IHCs at P10 and P22 (data not shown). Therefore, it is unlikely that vGlut3 expression was first initiated but not maintained in new IHCs. These observations support the conclusion that new IHCs were not fully differentiated even 4 months after they were formed.

Consistent with this, new IHCs had a high expression of EGFP in *PLP/CreER^T+^; Atoh1-HA+; α9AChR-EGFP+* mice at P22 and P90, and endogenous IHCs displayed a much weaker (at P22) or undetectable (at P90) EGFP expression (Fig. 4A–D). This observation indicates that new IHCs maintained the expression of α9AChR, and that in endogenous IHCs α9AChR expression decreased when they matured, as we have previously reported [39]. In addition, levels of calbindin were similar in new IHCs at P22 and P90, but were significantly decreased at P90 in endogenous and wild-type IHCs (Fig. 4E–H). Taken together, these results support the conclusion that the new IHCs were not able to fully differentiate.

Given the expression of otoferlin in new IHCs, we then analyzed the density of Ctbp2, a component of the pre-synaptic ribbons [45], [46], in 3 types of IHCs and 2 types of OHCs: 1) control IHCs in *Atoh1-HA+* control mice (cell circled in blue in Fig. 9B); 2) endogenous IHCs in *PLP/CreER^T+^; Atoh1-HA+* mice (cell circled in yellow in Fig. 9C); 3) new IHCs in *PLP/CreER^T+^; Atoh1-HA+* mice (cell circled in red in Fig. 9C); 4) control OHCs in *Atoh1-HA+* control mice (Fig. 9B); and 5) endogenous OHCs in *PLP/CreER^T+^; Atoh1-HA+* mice (Fig. 9C). We measured endogenous IHCs, adjacent to new IHCs, together with the endogenous OHCs, in the same cochleae of *PLP/CreER^T+^; Atoh1-HA+* mice (Fig. 9D). The number of Ctbp2+ puncta in new IHCs (11.7±0.5 puncta/IHC, *n* = 40, total cell number in 3 mice) was lower than that in control IHCs (15.6±0.3 puncta /IHC, *n* = 67) and endogenous IHCs (15.8±0.3, *n* = 79) but higher than that in control OHCs (2.5±0.1, *n* = 70) and endogenous OHCs (2.4±0.1, *n* = 69). We next determined whether new IHCs form functional ribbon synapses with neuronal fibers. We found that the pattern of innervation of new IHCs by the neuronal fibers visualized by either Tuj1 or neurofilament M labeling (Fig. 9E-E’’ and Fig. S4) was similar to that of endogenous IHCs. Finally, we examined the expression of the post-synaptic marker glutamate receptor type 2 (GluR2) near endogenous and new IHCs. Consistent with results of a previous study [47], pre-synaptic Ctbp2 and post-synaptic GluR2 puncta were closely aligned to each other in the IHC area in control samples (Fig. 9F-F’’’). In contrast, for the new IHCs, GluR2 signals were absent surrounding Ctbp2 puncta (Fig. 9G–I). Together, these results demonstrate that no functional synapses were formed between new IHCs and neuronal fibers, although neuronal innervation to new IHCs appeared normal. Because vGlut3 is required for IHC ribbon synapse maturation [42], [48], the lack of vGlut3 expression in new IHCs may explain the stalled synaptic maturation phenotypes we observed.

**Figure 10:**
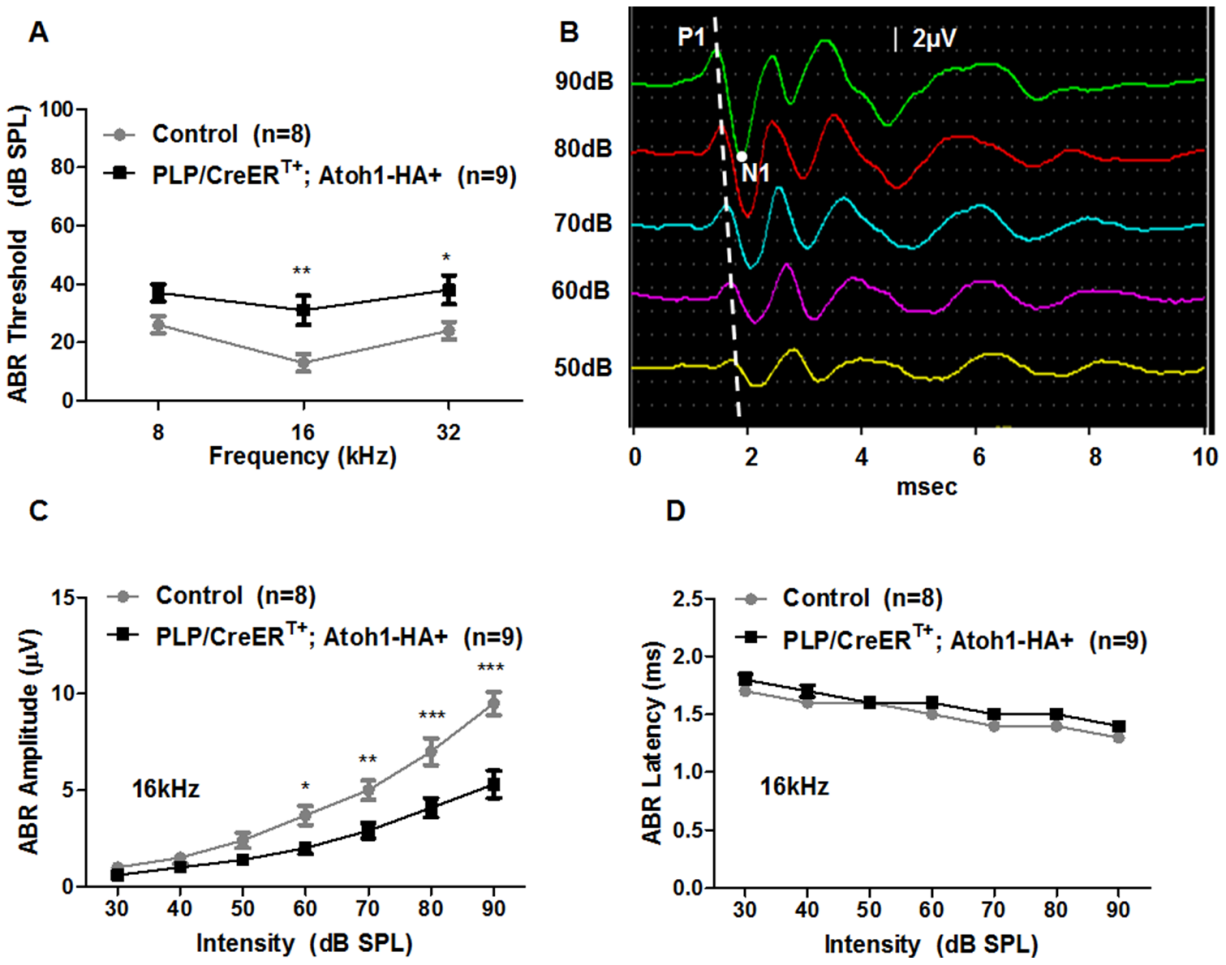
Comparison of auditory brainstem responses (ABRs) between control (grey line) and *PLP/CreER^T+^; Atoh1-HA+* (black line) mice. (A) ABR thresholds of 2 groups at 8, 16, and 32 kHz. (B) Representative of ABR recordings with wave1 peak positions (P1) (white dash line) and first negative peak N1 (white dot) positions. (C) ABR amplitude of wave1 (P1) at 16 kHz. (D) ABR peak latencies of wave1 (P1–N1) in the control (grey line) and *PLP/CreER^T+^; Atoh1-HA+* (black line) mice at 16 kHz. *P<0.05, **P<0.01, and ***P<0.001 as determined by a 2-way ANOVA followed by a Student's t-test with a Bonferroni correction.

We also measured auditory brainstem responses (ABR) in *PLP/CreER^T+^; Atoh1-HA+* and control mice. *PLP/CreER^T+^; Atoh1-HA+* mice had higher thresholds and lower amplitudes than those for control mice (Fig. 10A–C), whereas the ABR latencies of both groups were comparable (Fig. 10D). These results demonstrate that although the kinetics of synaptic transmission between endogenous IHCs and spiral ganglia was normal, the addition of new IHCs with no functional synapses had slight negative effects on the functionality of synaptic transmission between endogenous IHCs and spiral ganglia.

## Discussion

Our study showed that ectopic Atoh1 expression can efficiently convert neonatal IBs/IPhs into the specialized IHC fate, which can further differentiate into the Fgf8+/otoferlin+/vGlut3-negative immature IHC stage. Compared with previous studies on *in vivo* conversion in neonatal cochleae [8], our study provides new insights into the mechanism of *in vivo* conversion. Interestingly, we did not detect the expression of progenitor/stem cell markers (e.g., NeuroD1, Ngn1, Oct4 and Nanog) in IBs/IPhs soon after ectopic Atoh1 expression, suggesting that their conversion to HCs does not involve de-differentiation but rather direct cell fate conversion, a mechanism that is different from reprogramming between 2 highly differentiated cells via the intermediate state of induced pluripotent stem cells (iPS) or ES cells. Moreover, Pou4f3 was expressed in Atoh1+ IBs/IPhs before the completion of cell fate conversion, which is determined by the expression of HC markers (e.g., myosin and parvalbumin). In addition to its essential roles in HC survival [19], [49], recent studies suggest that Pou4f3 has a role in HC differentiation [50], [51]. This notion is supported by our finding that Pou4f3 expression precedes that of other HC markers in Atoh1-mediated cell fate conversion. As described in other systems during cell fate conversion, we observed a hybrid-state of transdifferentiating IBs/IPhs with the ectopic expression of Atoh1, most of which maintained the original IB/IPh characteristics (i.e., Sox2 expression) and gradually losing the IB/IPh molecular content (i.e., Glast1 expression).

Our findings highlight that IBs/IPhs are good candidates for regenerating IHCs *in situ.* This regeneration is in contrast to that of HCs derived from iPS or ES cells *in vitro*, in which a diverse range of basolateral voltage-dependent currents are observed [52], indicating the lack of specialization of new HCs. Moreover, HCs derived from DCs and PCs do not appear to adopt the specialized HC fate [8]. Our result that new HCs generated from IBs/IPhs differentiated further to adopt an IHC fate provides a unique opportunity to delineate the cell fate diversity between IHCs and OHCs during cochlear development and regeneration. The factors that contribute to the differential fates between IHCs and OHCs during development are currently not clear. Several key HC developmental genes such as *Atoh1, Gfi1, Pou4f3*, and *Barhl1* are expressed in both IHC and OHC lineages [53]–[57]. Consistent with previous studies in other SCs after ectopic Atoh1 expression [58], IBs/IPhs in our study were first converted into the primordial HC fate by the activation of multiple HC markers such as myosin VI/VIIa, parvalbumin, Lhx3, calbindin, calretinin, Espin, and α9AChR, although some of these markers become differentially expressed between IHCs and OHCs during their normal subsequent differentiation. Most strikingly, these new primordial HCs further preferred to adopt the IHC fate by developing the following unique IHC characteristics: 1) unique morphology of stereocilia bundles; 2) expression of Fgf8; 3) high-level expression of calretinin and otoferlin; 4) high density of Ctbp2 at synapses; and 5) large basolateral outward current amplitudes.

Why do IB/IPh-derived new HCs adopt IHC fate whereas DC/PC-derived HCs do not [8]? It is possible that the levels of Atoh1-HA expression are different in IBs/IPhs and DCs/PCs, as described previously [8], thus contributing to the IHC fate of IB/IPh-derived new HCs, as previously suggested [28]. It is also possible that after adopting the primordial HC fate in the HC lineage, the default cell fate is IHCs. This possibility is supported by the fact that in many genetic mutant mice IHCs were better maintained or their numbers were increased, although the total HC numbers were decreased [59], [60]. Moreover, IHCs are the primary mechanosensory receptors whereas OHCs are specific to mammals. Thus, additional steps may have been evolved in generating these more specialized OHCs. Therefore, we speculate that more signals or factors are needed to generate OHCs than to generate IHCs. This developmental guideline may apply to HC regeneration in postnatal cochleae. The modiolar region of the cochlea, also known as the greater epithelia ridge (GER), includes the Kolliker's organ, border cells, IHCs, and IBs/IPhs, whereas the lateral region of the cochlea, also known as the lesser epithelium ridge (LER), contains OHCs, PCs, DCs, and cells that eventually develop as the outer sulcus [61]. Cells in the GER and LER are not only morphologically distinct but also molecularly different [62], [63]. Because IBs/IPhs and IHCs are close to each other during development and both belong to the GER region medial to the organ of Corti [61], neonatal IBs/IPhs [61] may naturally adopt the preferred IHC fate in this permissive environment and contain unknown factors that, together with ectopic Atoh1, push IBs/IPhs on the initial track of IHC differentiation.

What factors influence the speed of cell fate conversion in vivo? When Atoh1 was ectopically expressed specifically in DCs and PCs in postnatal cochleae using a similar Atoh1 model, interestingly the conversion rate was not only low (∼6%) but also extremely slow, taking at least 3 weeks after induction [8]. In contrast, IPhs/IBs, after Atoh1 is ectopically turned on, need only 6 days to become HCs and the conversion rate reaches as high as 17.8% in three weeks. Therefore, IBs/IPhs may represent a source of stem/progenitor cells in the neonatal cochlea that can be turned into IHCs *in vivo*. The ability of Wnt to activate Atoh1 might also contribute to the competence to respond to Atoh1 ectopic expression [64]. Recently, overexpression of β-catenin in Lgr5+ SCs including IBs/IPhs resulted in proliferation of these SCs [65]. We recently showed that there was heterogeneity of Notch signaling among SCs during mouse cochlear development [66]. The SCs (i.e., IPhs/IBs) residing in the medial cochlea have lower Notch activities than those (PCs/DCs) in the lateral side. As Notch blocks transdifferentiation from SCs into HCs, the lower level of Notch in IBs/IPhs might contribute to the higher competence to respond to Atoh1 ectopic expression.

Although ectopic Atoh1 was able to convert a large number of IPhs/IBs into IHCs, these new IHCs retained some immature characteristics at both the molecular and functional levels. In fact, these new IHCS lacked vGlut3 expression, an IHC terminal differentiation marker. Moreover, new IHCs did not downregulate α9AChR as endogenous mature IHCs normally do [39]; exhibited lower density of Ctbp2 puncta and no GluR2 distribution at ribbon synapses than endogenous IHCs did despite the presence of neuronal innervation labeled by Tuj1 and neurofilament M; and displayed reduced, immature amplitudes of outward currents even at adult ages compared with those of mature endogenous IHCs. It is possible that constitutive Atoh1 expression in these new IHCs blocked their maturation, suggesting the importance of transient Atoh1 ectopic expression in HC regeneration [9]. As in many other systems where multiple factors, rather than a single factor, are required for efficient reprogramming *in vitro* or *in vivo* [67], it is possible that additional factors are needed to regenerate mature IHCs.

Our in vivo conversion of IPhs/IBs to IHCs by ectopic Atoh1 reported here is comparable with direct conversions reported in other regenerative systems. Neighboring cardiac fibroblasts can be converted to cardiomyocytes in vivo following ectopic expression of a combination of three to four cardiomyocyte transcription factors [68], [69]. Similarly, α islet cells can also be converted to β islet cells with ectopic expression of single pancreas transcription factor Pax4 [70]. In general, these in vivo conversions occur more efficiently than in vitro conversion following ectopic expression of similar factors, suggesting that in vivo factors promote regenerative efficiency. Therefore, *in vivo* cochlear environmental and intrinsic factors may provide the necessary natural clues for new IHCs to mature and fully differentiate. However, it is currently difficult to ablate or damage neonatal IHCs *in vivo* in combination with the use of Cre/LoxP for Atoh1 ectopic expression [71]. In addition, it remains unclear whether Atoh1 can convert adult IBs/IPhs into the IHC lineage, with or without prior IHC damage in the adult cochlea. Nonetheless, our current data support that targeting IBs/IPhs may represent a feasible therapeutic strategy to regenerate IHCs, as nonmammalian vertebrates have already evolved in using this effective regenerating strategy. Our success in generating IHCs *in vivo* highlights the importance of cell fate conversion in the nervous system and its potential application in neurodegenerative diseases.

## Materials and Methods

### Mouse Models

The generation of *CAG-loxP-stop-loxP-Atoh1-HA+* (Atoh1-HA+, founder #10) transgenic mouse line [8] and *CAG-loxP-stop-loxP-EGFP+* (requested from Dr. Jeffrey Robbins in Cincinnati Children's Hospital), *Rosa26-CAG-tdTomato^flox/+^*(# 007908 in The Jackson Laboratory), *α9AChR-EGFP+*, Fgf8-GFP mice (requested from Dr. Anne M. Moon in University of Utah), and *PLP/CreER^T^* mice (#005975 in The Jackson Laboratory) has been described previously [12], [25], [29], [39], [72]. Tamoxifen was injected intraperitoneally (IP) once daily at 3 mg/40 g in neonatal (P0 and P1) mice. FM 4-64FX dye (5 mg/kg, F34653, Invitrogen) was given to mice via subcutaneous injection. Mice of either sex were used for all experiments. The original *Atoh1-HA+* mice were maintained in a FVB background, and other mice were maintained as their original background (C57BL/6 or 129). The compound mice with multiple transgenes used in our study were therefore mixed backgrounds of C57BL/6, 129 and FVB. All animal experiments were approved by the Institutional Animal Care and Use Committee at St. Jude Children's Research Hospital.

### Tissue Preparation, Immunofluorescence, and Quantification Analysis

Tissue was prepared and examined as described previously [73], [74]. All images were taken by a confocal microscope (Zeiss LSM 700 or 710). To minimize the variation among mice, all the new HCs in each entire cochlea were quantified by confocal microscopy using the 40× lens, taking Z-stack images at a 1-µm interval. The cell number of Atoh1-HA+/Myosin VI+ cells was normalized to that of total Atoh1-HA+ cells (including new HCs derived from IBs/IPhs and the “presumptive” IBs/IPhs with Atoh1-HA expression) inside the organ of Corti to determine the percentage of IBs/IPhs that were converted into IHCs. The number of Sox2+/Atoh1-HA+/Myosin VI+ cells was normalized to the total number of Atoh1-HA+/Myosin VI+ cells to determine the percentage of new HCs that maintained Sox2 expression.

The following primary antibodies were used: anti-myosin VI (1∶200, cat#25–6791, Proteus Bioscience), anti-myosin VIIa (1∶200, cat#25-6790, Proteus Bioscience), anti-calbindin (1∶500, cat#AB1778, Millipore), anti-calretinin (1∶500, cat#AB5054, Millipore), anti-parvalbumin (1∶2000, cat#P3088, Sigma), anti-HA (1∶100, cat#11867431001, Roche), anti-prestin (1∶200, cat#sc-22692, Santa Cruz Biotechnology), anti-Lhx3 (1∶2000, cat#AB3202, Millipore), anti-GFP (1∶1000, cat#ab13970, Abcam), anti-Sox2 (1∶1000, cat#sc-17320, Santa Cruz Biotechnology), anti-Ctbp2 (1∶500, cat#612044, BD Transduction), anti-Pou4f3 (1∶500, cat#sc-81980, Santa Cruz Biotechnology), anti-NeuroD1 (1∶100, cat#sc-1084, Santa Cruz Biotechnology), anti-Ngn1 (1∶100, cat#PA5–11890, Thermo Scientific), anti-Oct4 (1∶100, cat#sc-9081, Santa Cruz Biotechnology), anti-Nanog (1∶100, cat#A300–398A, Bethyl Laboratories), anti-Neurofilament M (1∶100, cat#AB5735, Millipore), anti-Glast1 (1∶100, cat#ab416, Abcam), and anti-GluR2 (1∶200, cat#MAB397, Millipore), anti-Otoferlin (1∶100, cat#ab53233, Abcam) and anti-vGlut3 (1∶500, cat#135203, Synaptic Systems). All secondary antibodies were purchased from Invitrogen and used at a 1∶1000 dilution. Hoechst (1∶1000, H3570, Invitrogen) or ProLong® Gold Antifade Reagent with DAPI (P36941, Invitrogen) was used for counterstain nucleus.

### Fluorescence *in situ* Hybridization (FISH) Analysis

The copy number and chromosomal location of the Atoh1-HA line founder #10 [8] were determined by FISH analysis. Briefly, purified plasmid (5.5 kb Atoh1-HA) DNA was labeled with red-dUTP (Alexa Fluor 594, Molecular Probes) by nick translation. The labeled transgene probe and a green-dUTP (Abbott Molecular)–labeled chromosome 10 control probe (RP24–360A19 of 245 kb bacterial artificial chromosome or BAC) were combined with sheared mouse cot DNA and hybridized to metaphase and interphase nuclei derived from the transgenic mouse lung fibroblast cultures in a solution containing 50% formamide, 10% dextran sulfate, and 2× SSC. The chromosomes were then stained with 4,6-diamidino-2-phenylindole (DAPI) and analyzed. The Atoh1-HA intensity was consistent with ∼50 copies of the transgene, as determined by genomic Southern blot analysis.

### Fgf8 *in situ* Hybridization

*In situ* hybridization was performed as described previously [75], with the following modifications. Briefly, antisense RNA probes were generated from cDNA-containing plasmids and labeled with digoxigenin by *in vitro* transcription, using the DIG RNA labeling kit (Roche Applied Science, Cat# 11175025910). Whole-mount *in situ* hybridization was performed on mutant and corresponding control cochleae at various ages (P0, P3, P8, and P10). The 2% paraformaldehyde–fixed cochleae were digested briefly with 10 mg/mL of proteinase K for 5–10 min. Cochleae were hybridized with the Fgf8 probe overnight at 60°C in the hybridization solution. After washing, cochleae were incubated overnight at 4°C with an anti-digoxigenin antibody conjugated with alkaline phosphatase (Roche Applied Science, Cat# 11093274910). After several washes, cochleae were incubated in the dark with NBT/BCIP (BM purple substrate, Roche Applied Science, Cat# 11442074001). After periodic monitoring, the reaction was stopped by washing several times with KTBT. The organs of Corti were flat mounted and imaged.

### Electrophysiological Measurement

Electrophysiological features were studied on isolated cochleae at various ages by using the Olympus BX51 fluorescence microscope. Freshly prepared whole-mount cochleae were mounted in the recording chamber. Endogenous HCs were recognized by location, cell body shape, stereocilia shape, and EGFP (in α9-AChR-EGFP transgene), whereas newly formed HCs from IBs/IPhs were identified by EGFP and tdTomato (*PLP/CreER^T+^* **-**mediated) coexpression. HC currents were recorded by using the JClamp software (SciSoft, New Haven, CT) under a whole-cell voltage clamp configuration with an axoclamp 200B amplifier (Molecular Devices) coupled with a data acquisition board (National Instruments Corporation, Austin, TX).

To record the outward current, the bath solution contained 135 mM NaCl, 5.8 mM KCl, 1.3 mM CaCl_2_, 0.9 mM MgCl_2_, 0.7 NaH_2_PO_4_, 10 mM HEPES-NaOH, 5.6 mM D-glucose, and 2 mM sodium pyruvate. The pH was adjusted to 7.4 and the osmolality was approximately 300 mOsm. The pipette internal solution composition was 125 mM KCl, 5 mM Mg ATP, 10 mM HEPES, 5 mM creatine phosphate, 1 mM EGTA, and 2 mM ascorbate (pH 7.4, ∼300 mOsm). Patch pipettes were pulled by using a pipette puller (P2000, Sutter Instruments, Novato, CA). Recording pipette resistances were 2–3 MΩ in the extracellular solution. Currents were elicited by hyperpolarizing and depolarizing voltage steps (10 mV each step, –120 mV to +50 mV) from the holding potential of −84 mV. Only HCs that had a membrane resistance of more than 500 MΩ were included in the analysis.

### Statistical analyses

All data are expressed as mean ± SEM. ABR data were analyzed by two-way ANOVA. Cell quantification analysis was performed by using the Student's *t* test with a Bonferroni correction. GraphPad Prism 5.0 was used for all statistical analyses.

## Supporting Information

Figure S1 Fluorescence *in situ* hybridization of a metaphase mouse lung fibroblast cell from a heterozygous Atoh1-HA transgenic mouse showing that the Atoh1-HA transgene was inserted at a single site in chromosome 10 (band 10A3–4) of the mouse genome. Two pairs of green dots represent the control RP24-360A19 bacterial artificial chromosome probe (245 kb) conjugated with green-dUTP. One pair of red dots represents the Atoh1-HA transgenic probe (5.5 kb) conjugated with red-dUTP. DNA was counterstained with DAPI in blue. The arrow represents the chromosome 10 band 10A3–4 with Atoh1-HA transgene. The arrowhead labels the chromosome 10 band 10A3–4 without the Atoh1-HA transgene.
(TIF)

Figure S2 Atoh1-HA+ IBs/IPhs did not express progenitor or stem cell marker before cell fate conversion. Co-labeling of Atoh1-HA and NeuroD1 (A–A’’’), Ngn1 (B–B’’’), Oct4 (C–C’’’), Nanog (D–D’’’) in cochleae of *PLP/CreER^T+^; Atoh1-HA+* mice at P3, respectively. Inset in (A’’) and (B’’) are the positive control images taken in the same samples in spiral ganglion regions. Inset in (C’’) and (D’’) are the positive control images taken in mouse ES cells using the antibodies. Scale bar: 10 µm that applies to all panels.
(TIF)

Figure S3 Optical confocal image of FM4-64 FX dye and Atoh1-HA colabeling. Cochlea was dissected from *PLP/CreER^T+^; Atoh1-HA+* mice at P90 and transiently exposed to FM4-64FX dye for 30 s only. Arrows label the same new IHC. OHCs: outer hair cells; IHC: inner hair cell. Scale bars: 20 µm.
(TIF)

Figure S4 Optical confocal images of co-staining of Myosin VIIa, Atoh1-HA, Ctbp2, and neurofilament M in *PLP/CreER^T+^; Atoh1-HA+* mice at P21. Both endogenous IHC (arrows in A–A’’’’) and new IHC (arrows in B–B’’’’) are innervated by neuronal fibers. Scale bars: 10 µm.
(TIF)
