# Supplementary figures and images for: In Vivo Generation of Immature Inner Hair Cells in Neonatal Mouse Cochleae by Ectopic Atoh1 Expression
Source: PLoS One. 2014 Feb 20;9(2):e89377. doi: 10.1371/journal.pone.0089377 (PMC3930725; doi:10.1371/journal.pone.0089377)

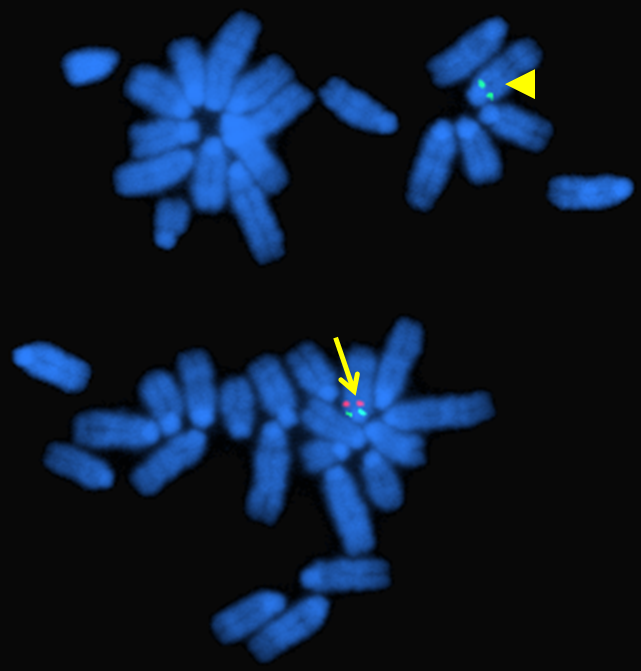

Supplement: Figure S1 — Fluorescence in situ hybridization of a metaphase mouse lung fibroblast cell from a heterozygous Atoh1-HA transgenic mouse showing that the Atoh1-HA transgene was inserted at a single site in chromosome 10 (band 10A3–4) of the mouse genome. Two pairs of green dots represent the control RP24-360A19 bacterial artificial chromosome probe (245 kb) conjugated with green-dUTP. One pair of red dots represents the Atoh1-HA transgenic probe (5.5 kb) conjugated with red-dUTP. DNA was counterstained with DAPI in blue. The arrow represents the chromosome 10 band 10A3–4 with Atoh1-HA transgene. The arrowhead labels the chromosome 10 band 10A3–4 without the Atoh1-HA transgene. (TIF) [file pone.0089377.s001.tif]

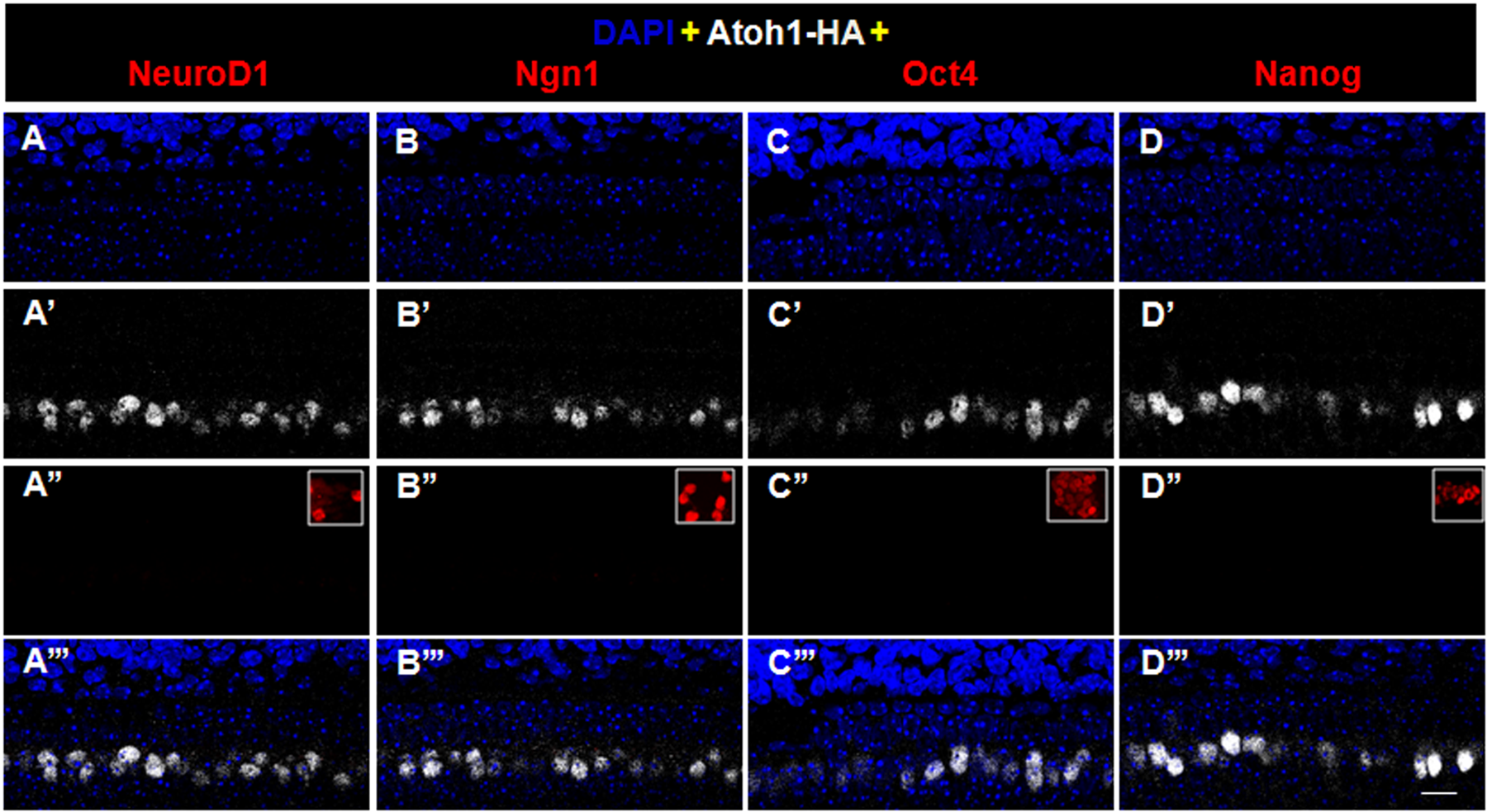

Supplement: Figure S2 — Atoh1-HA+ IBs/IPhs did not express progenitor or stem cell marker before cell fate conversion. Co-labeling of Atoh1-HA and NeuroD1 (A–A’’’), Ngn1 (B–B’’’), Oct4 (C–C’’’), Nanog (D–D’’’) in cochleae of PLP/CreERT+; Atoh1-HA+ mice at P3, respectively. Inset in (A’’) and (B’’) are the positive control images taken in the same samples in spiral ganglion regions. Inset in (C’’) and (D’’) are the positive control images taken in mouse ES cells using the antibodies. Scale bar: 10 µm that applies to all panels. (TIF) [file pone.0089377.s002.tif]

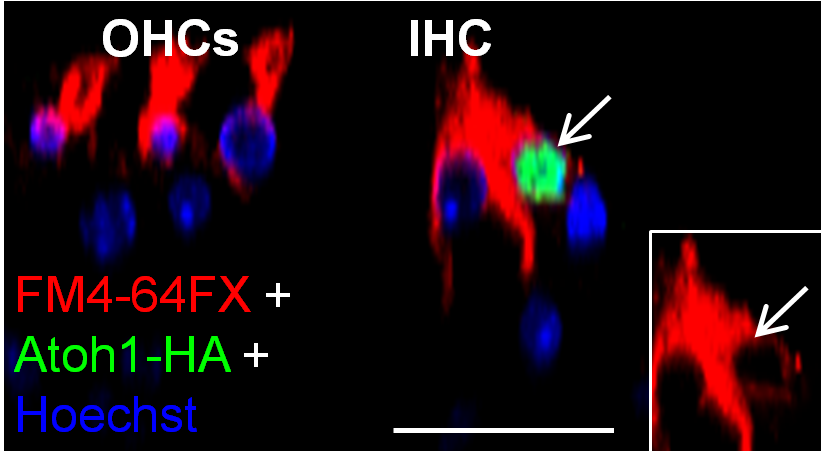

Supplement: Figure S3 — Optical confocal image of FM4-64 FX dye and Atoh1-HA colabeling. Cochlea was dissected from PLP/CreERT+; Atoh1-HA+ mice at P90 and transiently exposed to FM4-64FX dye for 30 s only. Arrows label the same new IHC. OHCs: outer hair cells; IHC: inner hair cell. Scale bars: 20 µm. (TIF) [file pone.0089377.s003.tif]

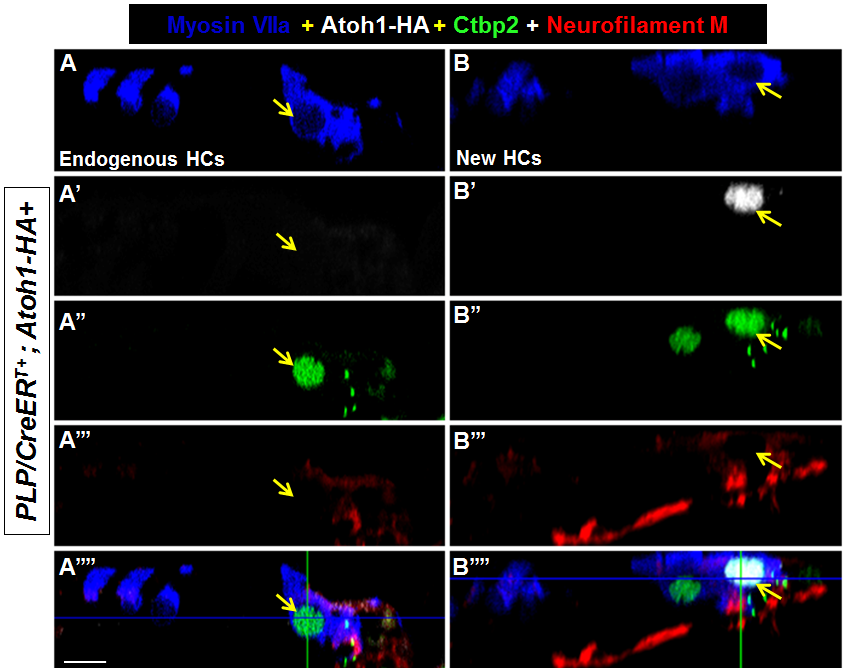

Supplement: Figure S4 — Optical confocal images of co-staining of Myosin VIIa, Atoh1-HA, Ctbp2, and neurofilament M in PLP/CreERT+; Atoh1-HA+ mice at P21. Both endogenous IHC (arrows in A–A’’’’) and new IHC (arrows in B–B’’’’) are innervated by neuronal fibers. Scale bars: 10 µm. (TIF) [file pone.0089377.s004.tif]
